# Risk factors for peripheral venous catheter failure: A prospective cohort study of 5345 patients

**DOI:** 10.1177/11297298211015035

**Published:** 2021-05-13

**Authors:** Ya-mei Chen, Xiao-wen Fan, Ming-hong Liu, Jie Wang, Yi-qun Yang, Yu-fang Su

**Affiliations:** 1 Department of Emergency, The First Affiliated Hospital of Soochow University, Suzhou, China; 2 Nursing College, Soochow University, Suzhou, China; 3 Department of Hematology, The First Affiliated Hospital of Soochow University, Suzhou, China; 4 Department of Nursing, The First Affiliated Hospital of Soochow University, Medical Centre of Soochow University, Suzhou, China; 5 Department of Orthopaedic, The First Affiliated Hospital of Soochow University, Suzhou, China

**Keywords:** Peripheral venous catheter, complication, risk factors, prediction model

## Abstract

**Purpose:**

The objective of this study was to determine the independent risk factors associated with peripheral venous catheter (PVC) failure and develop a model that can predict PVC failure.

**Methods:**

This prospective, multicenter cohort study was carried out in nine tertiary hospitals in Suzhou, China between December 2017 and February 2018. Adult patients undergoing first-time insertion of a PVC were observed from catheter insertion to removal. Logistic regression was used to identify the independent risk factors predicting PVC failure.

**Results:**

This study included 5345 patients. The PVC failure rate was 54.05% (*n* = 2889/5345), and the most common causes of PVC failure were phlebitis (16.3%) and infiltration/extravasation (13.8%). On multivariate analysis, age (45–59 years: OR, 1.295; 95% CI, 1.074–1.561; 60–74 years: OR, 1.375; 95% CI, 1.143–1.654; ⩾75 years: OR, 1.676; 95% CI, 1.355–2.073); department (surgery OR, 1.229; 95% CI, 1.062–1.423; emergency internal/surgical ward OR, 1.451; 95% CI, 1.082–1.945); history of venous puncture in the last week (OR, 1.298, 95% CI 1.130–1.491); insertion site, number of puncture attempts, irritant fluid infusion, daily infusion time, daily infusion volume, and type of sealing liquid were independent predictors of PVC failure. Receiver operating characteristic curve analysis indicated that a logistic regression model constructed using these variables had moderate accuracy for the prediction of PVC failure (area under the curve, 0.781). The Hosmer-Lemeshow goodness of fit test demonstrated that the model was correctly specified (χ^2^ = 2.514, *p* = 0.961).

**Conclusion:**

This study should raise awareness among healthcare providers of the risk factors for PVC failure. We recommend that healthcare providers use vascular access device selection tools to select a clinically appropriate device and for the timely detection of complications, and have a list of drugs classified as irritants or vesicants so they can monitor patients receiving fluid infusions containing these drugs more frequently.

## Introduction

Peripheral venous catheter (PVC) is commonly used to obtain short-term venous access and administer intravenous therapy. PVC insertion is among the most frequent invasive procedures performed by healthcare providers.^1,2^ Although PVC has widespread use, an estimated 26%–69% of PVCs fail,^3–5^ mainly due to complications such as phlebitis, occlusion, dislodgement, infiltration/extravasation, and local infection.^6,7^ PVC failure is associated with interruption of treatment, catheter replacements, infection and mortality, and can increase length of hospital stay and healthcare costs.^8,9^ Therefore, there remains an unmet clinical need to identify the risk factors associated with PVC failure and prevent catheter complications.

Previous studies have confirmed that patient age,^10^ catheter type,^3,11^ insertion site,^12^ puncture technique,^3^ and chronic disease^13^ affect the incidence of PVC failure. However, these studies were limited by small sample size and their retrospective design, which may bias the results. In addition, predictive models of PVC failure in adult patients are scarce. A prediction model will facilitate early identification of PVC failure, enable timely intervention, and reduce the clinical and economic burden of catheter complications.

The objective of this study was to determine the independent risk factors associated with PVC failure and develop a model that can predict PVC failure. This model may have clinical utility for early identification of PVC failure.

## Methods

### Study design, and participants

**Figure 1.**
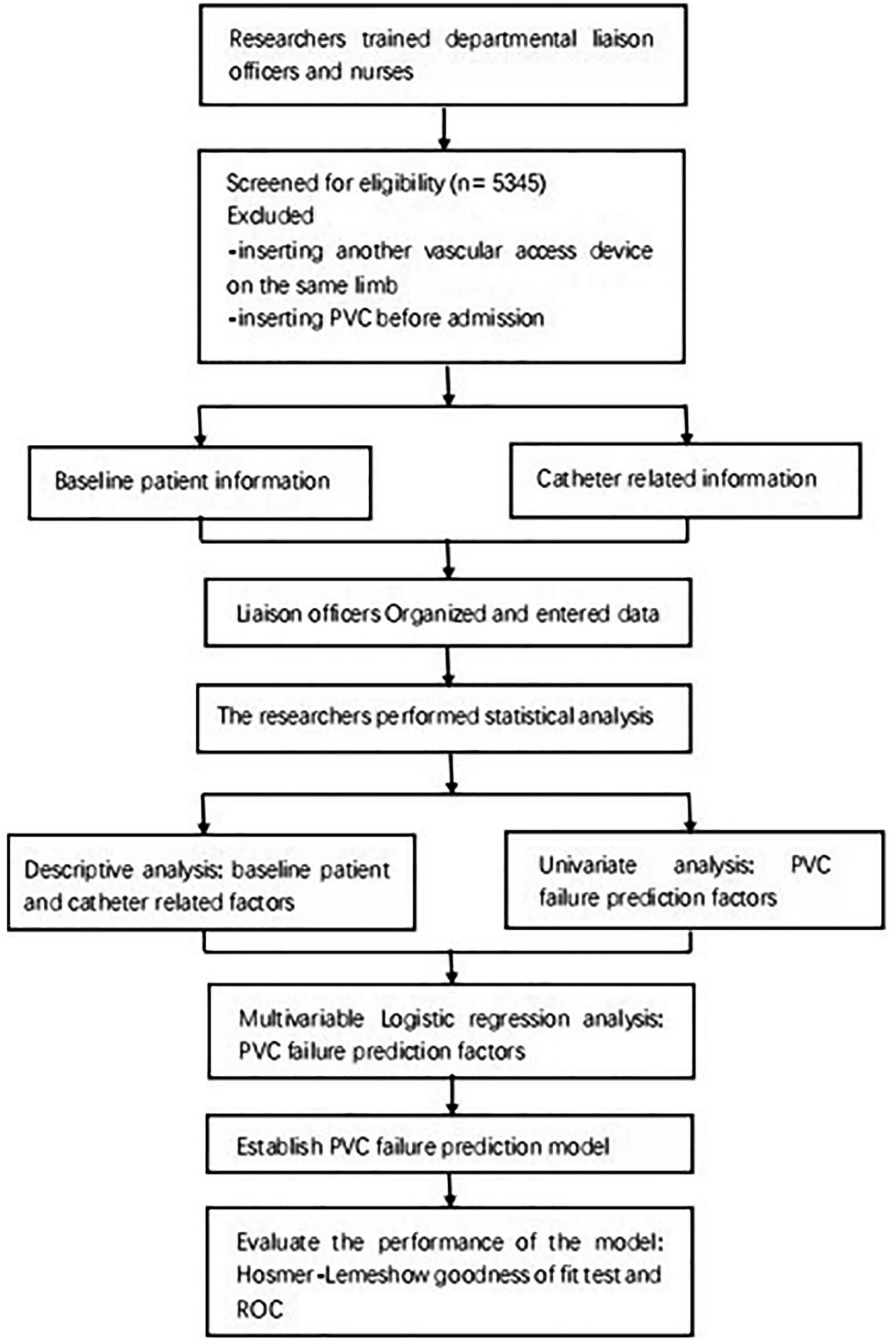
Flow chart of study design.

The study design is shown in Figure 1. Patients who were hospitalized in nine tertiary hospitals in Suzhou, China between December 2017 and February 2018 were eligible to participate in this prospective multicenter cohort study. Inclusion criteria were (1) aged ⩾18 years and (2) undergoing first-time insertion of a PVC. Exclusion criteria were (1) insertion of another vascular access device into the same limb or (2) insertion of a PVC before admission. All patients provided written informed consent.

### Sampling and sample size

This study used cluster sampling. The following formula was used to calculate the required sample size: *n* = μ^2^_α/2_*P*(1 − *P*)/δ^2^ where *n* is the sample size, μ is the population mean, α is the Type I error probability, *P* is the estimated PVC failure rate, and δ is the allowable error. Assuming that 26% of PVCs fail,^5^ the calculated sample size was 1094. Considering a loss to follow up rate of 20%, the sample size for this study was 1313.

### Data collection

Patient recruitment and data collection were performed by nurses. Data were organized and recorded by liaison officers. The lead author trained the nurses and liaison officers, supervised compliance with the study protocol, confirmed data integrity, and performed data analysis. All insertion, maintenance, and removal of PVCs were performed by qualified nurses, in accordance with standardized procedures.

Data collection included patient baseline demographic and clinical characteristics, such as age, gender, department, admitting diagnosis and comorbidities, and catheter related-information such as insertion site, irritant infusion, securement method, dressing type, infusion regimen, daily infusion time, and causes of PVC failure. The insertion site was evaluated at shift change or if there were signs of PVC failure. The PVC was removed at the end of treatment, due to complications, or 72–96 h after insertion.

PVC failure was defined as catheter removal for any reason other than exceeding the maximum indwelling time or completion of infusion therapy. Reasons for PVC failure included, but were not limited to, phlebitis, infiltration/extravasation, occlusion, dislodgement, or leakage from the insertion site. Phlebitis was assessed using the Infusion Nurses Society (INS) phlebitis scale.^14^ Infiltration/extravasation was defined as leakage of an irritant or vesicant from the blood vessels to surrounding tissues.^15^ Occlusion was defined as the inability to infuse intravenous fluids. Leakage from the insertion site was defined as inadvertent leakage of intravenous fluids from the insertion site. Dislodgement was defined as accidental removal or movement of the device that resulted in loss of function.

### Statistical analysis

Data were analyzed using SPSS18.0 statistical software. Normally distributed data are reported as mean and standard deviation. Non-normally distributed data are reported as medians and interquartile ranges. Categorical data are reported as frequencies and proportions. The frequency of PVC failure was calculated. Univariate and multivariate logistic regression analyses were used to determine predictors of PVC failure. Univariate analysis used the chi-square test. Statistically significant variables (*p* < 0.05) were included in the multivariate analysis. Odds ratios (OR) and their corresponding 95% confidence intervals (CI) were calculated. Statistically significant variables were used to establish a predictive model. Receiver operating characteristic curve (ROC), area under the curve (AUC; where 0.5 = chance result; 0.5–0.7 = low accuracy; 0.7–0.9 = moderate accuracy; 0.9 = high accuracy), and the Hosmer-Lemeshow goodness of fit test were used to evaluate model performance.

## Results

This study included 5345 patients hospitalized on wards associated with the following departments: internal medicine (digestive, respiratory, cardiology, neurology), surgery (general surgery, orthopedics, thoracic surgery, neurosurgery) and obstetrics and gynecology, and on an emergency internal/surgical ward that included patients with acute pancreatitis, gastrointestinal bleeding, pulmonary infection, multiple trauma, and cerebral hemorrhage.

Patient baseline demographic and clinical characteristics are summarized in Table 1. Median age of included patients was 59 years (range, 18–102), and 46.4% (2481) of patients were male. Most patients were hospitalized on internal medicine wards (2081, 38.9%) and surgery wards (2144, 40.1%). 31.4% (*n* = 1680) of the patients had hypertension and diabetes.

**Table 1.** Patient baseline demographic and clinical characteristics.

Variable	PVC failure		Overall (*N* = 5345), No. (%)	χ^2^	*p*	OR (95% CI)
	Yes (*N* = 2889; 54.05), No. (%)	No (*N* = 2456; 45.95), No. (%)				
Gender						
Male	1363 (47.2)	1118 (45.5)	2481 (46.4)	1.467	0.226	—
Female	1526 (52.8)	1338 (54.5)	2864 (53.6)			1.069 (0.960, 1.191)
Age						
⩽44	729 (25.2)	789 (32.1)	1518 (28.4)	35.024	<0.001	—
45–59	670 (23.2)	533 (21.8)	1203 (22.5)			1.360 (1.169, 1.584)
60–74	902 (31.2)	728 (29.6)	1630 (30.5)			1.341 (1.165, 1.543)
⩾75	588 (20.4)	406 (16.5)	994 (18.6)			1.567 (1.334,1.843)
Department						
Internal medicine	1071 (37.1)	1010 (41.1)	2081 (38.9)	102.255	<0.001	—
Surgery	1284 (44.4)	860 (35.0)	2144 (40.1)			1.408 (1.246, 1.590)
Obstetrics and gynecology	334 (11.6)	476 (19.4)	810 (15.2)			0.662 (0.562, 0.780)
Emergency internal/surgical ward	200 (6.9)	110 (4.5)	310 (5.8)			1.715 (1.338, 2.197)
Comorbidities						
No	1947 (67.4)	1703 (69.3)	3650 (68.3)	2.782	0.595	—
Diabetes	139 (4.8)	111 (4.5)	250 (4.7)			1.095 (0.846, 1.417)
Hypertension	184 (25.0)	522 (21.3)	1183 (22.1)			1.108 (0.971, 1.264)
Diabetes and hypertension	661 (22.9)	114 (4.6)	247 (4.6)			1.020 (0.788, 1.321)
Other	9 (0.3)	6 (0.3)	15 (0.3)			1.312 (0.466, 3.694)
History of venous puncture for nearly 1 week						
No	1962 (67.9)	1809 (73.7)	3771 (70.6)	21.078	<0.001	—
Yes	927 (32.1)	647 (26.3)	1574 (29.4)			1.321 (1.173, 1.488)
Catheter type						
Closed normal type	2025 (70.1)	1705 (69.4)	3730 (69.8)	8.092	0.017	—
Closed safety type	814 (28.2)	680 (27.7)	1494 (28.0)			1.008 (0.893, 1.137)
Open type	50 (1.7)	71 (2.9)	121 (2.3)			0.593 (0.411, 0.856)
Infusion tap type						
Heparin lock	2729 (94.5)	2354 (95.8)	5083 (95.1)	5.464	0.019	—
Needleless connector	160 (5.5)	102 (4.2)	262 (4.9)			1.353 (1.049, 1.745)
Gauge						
24 g	2038 (70.5)	1648 (67.1)	3686 (69.0)	7.369	0.025	—
22 g	712 (24.6)	674 (27.4)	1386 (25.9)			0.854 (0.755, 0.967)
18–20 g	139 (4.8)	134 (5.5)	273 (5.1)			0.839 (0.656, 1.073)
Insertion site						
Back of hand	1425 (49.3)	1352 (55.0)	2777 (52.0)	23.102	<0.001	—
Wrist	229 (7.9)	199 (8.1)	428 (8.0)			1.092 (0.890, 1.339)
Forearm	1123 (38.9)	834 (34.0)	1957 (36.6)			1.278 (1.137, 1.435)
Antecubital fossa	70 (2.4)	52 (2.1)	122 (2.3)			1.277 (0.885, 1.842)
Other	42 (1.5)	19 (0.8)	61 (1.1)			2.097 (1.214, 3.624)
Number of puncture attempts						
1	2662 (92.1)	2322 (94.5)	4984 (93.2)	12.155	<0.001	—
⩾2	227 (7.9)	134 (5.5)	361 (6.8)			1.478 (1.185, 1.842)
Securement regimen						
Transparent dressings	2869 (99.3)	2429 (98.9)	5298 (99.1)	3.098	0.377	—
Gauze+ adhesive tape	8 (0.30)	10 (0.40)	18 (0.3)			0.677 (0.267, 1.719)
Disposable wound dressing	1 (0.10)	3 (0.10)	4 (0.1)			0.282 (0.029, 2.715)
Transparent dressings+ gauze	11 (0.40)	14 (0.60)	25 (0.5)			0.665 (0.301, 1.468)
Dressing type						
Ordinary	1225 (42.4)	778 (31.7)	2003 (37.5)	65.164	<0.001	—
Reinforced	1664 (57.6)	1678 (68.3)	3342 (62.5)			0.630 (0.563, 0.705)
Irritant fluid infusion						
No	2027 (70.2)	2016 (82.1)	4043 (75.6)	102.402	<0.001	—
Yes	862 (29.8)	440 (17.9)	1302 (24.4)			1.948 (1.710, 2.220)
Infusion apparatus type						
Ordinary	2059 (71.3)	1753 (71.4)	3812 (71.3)	0.007	0.932	—
Precise	830 (28.7)	703 (28.6)	1533 (28.7)			1.005 (0.892, 1.132)
Infusion regimen						
Intermittent therapy	2799 (96.9)	2410 (98.1)	5209 (97.5)	8.262	0.004	—
Continuous therapy	90 (3.1)	46 (1.9)	136 (2.5)			1.685 (1.176, 2.413)
Daily infusion time						
<4 h	750 (26.0)	1029 (41.9)	1779 (33.3)	163.160	<0.001	—
4–6 h	1275 (44.1)	930 (37.9)	2205 (41.3)			1.881 (1.657, 2.135)
>6 h	781 (27.0)	455 (18.5)	1236 (23.1)			2.355 (2.029, 2.734)
Daily infusion volume						
⩽500	695 (24.1)	944 (38.4)	1639 (30.7)	141.49	<0.001	—
501–1000	1262 (43.7)	950 (38.7)	2212 (41.4)			1.804 (1.586, 2.053)
1001–1500	498 (17.2)	323 (13.2)	821 (15.4)			2.094 (1.765, 2.484)
>1500	434 (15.0)	239 (9.7)	673 (12.6)			2.466 (2.048, 2.970)
Flushing before infusion						
No	59 (2.0)	4 (0.2)	63 (1.2)	40.254	<0.001	—
Yes	2830 (98.0)	2452 (99.8)	5282 (98.8)			0.078 (0.028, 0.216)
Flushing fluid						
Saline	2745 (95.0)	2377 (96.8)	5122 (95.8)	40.267	<0.001	—
Heparin saline	85 (2.9)	75 (3.1)	160 (3.0)			0.981 (0.716, 1.345)
Flushing fluid volume						
⩽5	2707 (93.7)	2363 (96.2)	5070 (94.9)	42.007	<0.001	—
>5	123 (4.3)	89 (3.6)	212 (4.0)			1.206 (0.913, 1.593)
Sealing regimen						
Positive pressure sealing tube	2833 (98.1)	2456 (100.0)	5289 (99.0)	69.412	<0.001	—
Use positive pressure connector without sealing tube	55 (1.9)	0 (0.0)	55 (1.0)			0.000
Sealing liquid						
Saline	2736 (94.7)	2408 (98.0)	5144 (96.2)	58.343	<0.001	—
Heparin saline	98 (3.4)	48 (2.0)	146 (2.7)			1.797 (1.267, 2.549)
Sealing liquid volume						
⩽5	2751 (95.2)	2379 (96.9)	5130 (96.0)	47.434	<0.001	—
>5	83 (2.9)	77 (3.1)	160 (3.0)			0.932 (0.680, 1.277)
Indwelling time						
*t* ⩽24 h	529 (18.3)	165 (6.7)	694 (13.0)	873.388	<0.001	—
24 h < *t* ⩽ 48 h	1001 (34.6)	332 (13.5)	1333 (25.0)			0.940 (0.759, 1.165)
48 h < *t* ⩽ 72 h	896 (31.0)	720 (29.3)	1616 (30.2)			0.388 (0.318, 0.474)
72 h < *t* ⩽ 96 h	328 (11.4)	936 (38.2)	1264 (23.6)			0.109 (0.088, 0.136)
*t* > 96h	135 (4.7)	303 (12.3)	438 (8.2)			0.139 (0.106, 0.182)

Reasons for PVC failure are summarized in Table 2. The PVC failure rate was 54.05% (*n* = 2889/5345). The most common causes of PVC failure were phlebitis (16.3%) and infiltration/extravasation (13.8%).

**Table 2.** Causes of PVC failure.

	*N*	% of Total (*n* = 5345)	
Reasons for PVC failure			% *F* (*n* = 2889)
Phlebitis	873	16.3	873
Infiltration/extravasation	737	13.8	737
Occlusion	654	12.2	654
Local liquid leakage	362	6.8	362
Dislodgement	36	0.7	36
Changed to CVC	55	0.1	55
Prevention of complications	84	1.6	84
Patient’s request	69	1.3	69
Operation	19	0.4	19
Reasons for PVC removal			% PIVC removal (*n* = 2456)
No longer required	1521	28.5	1521
Routine removal at 72 h	935	17.5	935

CVC: central venous catheter.

### Multivariate analysis

Multivariate analysis of predictors of PVC failure are summarized in Table 3. On multivariate analysis, age (45–59 years: OR, 1.295; 95% CI, 1.074–1.561; 60–74 years: OR, 1.375; 95% CI, 1.143–1.654; ⩾75 years: OR, 1.676; 95% CI, 1.355–2.073), department (surgery OR, 1.229; 95% CI, 1.062–1.423; emergency internal/surgical ward OR, 1.451; 95% CI, 1.082–1.945), history of venous puncture in the last week (OR, 1.298, 95% CI 1.130–1.491), insertion site (forearm: OR, 1.201; 95% CI, 1.045–1.380), number of puncture attempts (>2: OR, 1.317; 95% CI, 1.025–1.693), irritant fluid infusion (OR, 1.344, 95% CI, 1.148–1.574), daily infusion time (4–6 h: OR, 1.513; 95% CI, 1.264–1.810; >6 h: OR, 1.868; 95% CI 1.479–2.359), daily infusion volume (501–1000: OR, 1.328; 95% CI, 1.108–1.592), and type of sealing liquid (heparinized saline: OR, 1.521; 95% CI 1.023–2.261) were independent predictors of PVC failure. Department (obstetrics and gynecology: OR, 0.513; 95% CI, 0.407–0.647); catheter type (closed safety PVC: OR, 0.824; 95% CI, 0.713–0.952; open PVC: OR, 0.359; 95% CI, 0.226–0.571); reinforced dressing (OR, 0.65; 95% CI, 0.569–0.742); and indwelling period (48 h < *t* ⩽72 h: OR, 0.405; 95% CI, 0.327–0.502; 72 h < *t* ⩽ 96 h: OR, 0.107; 95% CI, 0.085–0.135; *t* > 96 h: OR, 0.126; 95% CI, 0.094–0.167) were independent predictors of PVC success.

**Table 3.** Logistic regression analysis of predictors of PVC failure.

Variable	β	Standard error	Wald	*p*	OR	95% CI
Constant	0.453	0.148	9.374	0.002	0.453	
Age						
⩽44			23.255	0.000		
45–59	0.258	0.096	7.31	0.007	1.295	1.074–1.561
60 74	0.318	0.094	11.41	0.001	1.375	1.143–1.654
⩾75	0.516	0.108	22.676	0.000	1.676	1.355–2.073
Department						
Internal medicine			67.565	0.000		
Surgery	0.207	0.074	7.690	0.006	1.229	1.062–1.423
Obstetrics and gynecology	−0.668	0.118	31.892	0.000	0.513	0.407–0.647
Emergency internal/surgical ward	0.372	0.150	6.199	0.013	1.451	1.082–1.945
History of venous puncture for nearly 1 week						
No				1		
Yes	0.261	0.071	13.652	0.000	1.298	1.130–1.491
Catheter type						
Closed normal type			23.435	0.000		
Closed safety type	−0.194	0.074	6.941	0.008	0.824	0.713–0.952
Opened type	−1.023	0.236	18.802	0.000	0.359	0.226–0.571
Insertion site						
Back of hand			13.133	0.011		
Wrist	0.165	0.121	1.862	0.172	1.179	0.931–1.493
Forearm	0.183	0.071	6.657	0.010	1.201	1.045–1.380
Antecubital fossa	0.061	0.212	0.082	0.774	1.063	0.702–1.608
Number of puncture attempts						
1				1		
⩾2	0.276	0.128	4.633	0.031	1.317	1.025–1.693
Dressing type						
Ordinary				1		
Reinforced	−0.431	0.068	40.755	0.000	0.65	0.569–0.742
Irritant fluid infusion						
No				1		
Yes	0.296	0.08	13.519	0.000	1.344	1.148–1.574
Daily infusion time						
<4 h			30.846	0.000		
4–6 h	0.414	0.092	20.355	0.000	1.513	1.264–1.810
>6 h	0.625	0.119	27.535	0.000	1.868	1.479–2.359
Daily infusion volume						
⩽500			10.437	0.015		
500–1000	0.284	0.093	9.418	0.002	1.328	1.108–1.592
1001–1500	0.209	0.127	2.703	0.100	1.232	0.961–1.580
>1500	0.113	0.146	0.597	0.440	1.120	0.840–1.492
Sealing liquid						
Saline				1.000		
Heparin saline	0.419	0.202	4.296	0.038	1.521	1.023–2.261
Indwelling time						
*t* ⩽ 24 h			732.212	0.000		
24 h < *t* ⩽ 48 h	−0.010	0.116	0.008	0.929	0.990	0.789–1.241
48 h < *t* ⩽ 72 h	−0.093	0.109	68.655	0.000	0.405	0.327–0.502
72 h < *t* ⩽ 96 h	−2.235	0.118	361.555	0.000	0.107	0.085–0.135
*t* > 96 h	−2.073	0.146	201.475	0.000	0.126	0.094–0.167

### Logistic regression model evaluation

**Figure 2.**
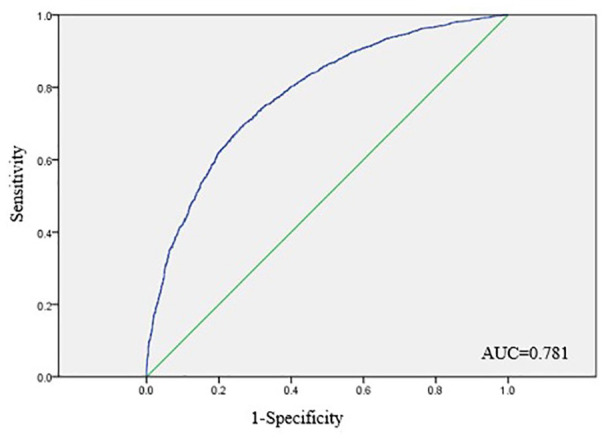
ROC curve analysis of the prective model of PVC failure.

ROC curve analysis indicated that the logistic regression model constructed using the variables identified on multivariate analysis had moderate accuracy for the prediction of PVC failure (AUC, 0.781) (Figure 2). The Hosmer-Lemeshow goodness of fit test demonstrated that the model was correctly specified (χ^2^ = 2.514, *p* = 0.961).

## Discussion

The objective of this study was to determine the independent risk factors associated with PVC failure and develop a model that can predict PVC failure in adult patients. In this study, 54.05% of PVCs inserted in adult patients failed. Predictors of PVC failure included older age, hospitalization on the surgery or emergency/internal/surgical ward, history of venous puncture in the last week, forearm insertion site, ⩾2 puncture attempts, irritant fluid infusion, daily infusion time >4 h, daily infusion volume 501–1000 ml, and use of heparinized saline as the sealing liquid. ROC curve analysis indicated that the logistic regression model constructed using these variables had moderate accuracy for the prediction of PVC failure, while the Hosmer-Lemeshow goodness of fit test demonstrated that the model was correctly specified.

Previous reports have described lower rates of PVC failure than the 54.05% reported in our study. Specifically, catheter failure occurred in 31% of patients with emergency department (ED)-inserted PVCs admitted to hospital wards in two large academically affiliated hospitals in Perth, Western Australia and in 32% of adult patients requiring a PVC on the medical and surgical wards of a tertiary hospital in Queensland, Australia.^10,16^ The disparate results between our study and the previous studies may be related to differences in the included patient populations. In the present study, almost 50% of the patients were aged ⩾60 years; therefore, vascular aging, which affects vascular stiffness and permeability, may have contributed to the high incidence of PVC failure.

In our study, the most common causes of PVC failure were phlebitis (16.3%) and infiltration/extravasation (13.8%). The incidence of phlebitis was lower than previous reports of PVC-related phlebitis in adult patients in tertiary hospitals in Croatia (44%)^17^ or in patients treated as urgent cases in EDs in Italy (31%),^18^ and infiltration and occlusion combined were the most common causes of PVC failure (47%) in the study of patients with ED-inserted PVCs conducted in Western Australia.^10^ Differences in the findings from our study and the previous studies may be attributed to differences in the characteritics of the patient populations or healthcare settings in which they were conducted, and the lack of a concencus definition and standard assessment tool for phlebitis, which makes diagnosis of phlebitis a challenge.^19^

Our study identified several factors as predictors of PVC failure in adult patients. Specifically, findings showed that the risk of PVC failure increased with increasing patient age. Consistent with these data, the risk of PVC failure was associated with being an older patient in the study of patients with ED-inserted PVCs conducted in Western Australia.^10^ Age-related impairment of vascular structure and function may lead to vascular stiffness, endothelial dysfunction, and hypoperfusion.

Our results also revealed that department was an independent predictor of PVC failure, whereby patients hospitalized on the surgery or emergency internal/surgical ward, mostly suffering from acute pancreatitis, gastrointestinal bleeding, pulmonary infection, multiple trauma, or cerebral hemorrhage, were more likely to experience PVC failure than patients hospitalized on other wards. In accordance with these findings, PVC failure was significantly associated with department (internal medicine, general surgery, orthopedics, gynecology, other surgery) in a previous study^20^ of adult patients scheduled for infusion therapy at one of two tertiary hospitals in Hunan China. Patients on surgery or emergency internal/surgical wards often undergo abrupt changes in their clinical status and generally receive large infusions of fluid for fluid replacement and to deliver nutritional elements and medication. Infusion of large volumes of fluid can cause vascular damage and increase vascular permeability, which may induce PVC failure.

Our study suggested that patients with a history of venous puncture in the last week were more likely to experience PVC failure. Consistent with these data, a study of patients with PVCs on the admission units and intermediate care unit of a second level regional hospital of the Principality of Asturias Health Service identified previous insertion in the same arm as a risk factor for PVC failure.^21^ Multiple catheterizations at the same site may directly damage the vascular endothelial cells at that puncture site, causing local vasoconstriction, backflow of infusion fluid to the initial venous puncture site,^22^ and leakage of fluid from the blood vessels into the surrounding tissues.

Our results revealed that use of an insertion site in the forearm increased the risk of PVC failure. In accordance with these findings, a study of adult patients admitted to various wards at King Abdulaziz Medical City, Riyadh, Saudi Arabia demonstrated that phlebitis was predicted by PVC insertion in the fore/upper arm.^11^ In contrast, the INS guidelines^14^ state that forearm insertion may prolong catheter indwelling time, relieve pain, facilitate patient self-management, and prevent unplanned removal and occlusion; however, the strength of the body of evidence supporting these practice criteria is low. Inserting a PVC in the back of the hand may improve the likelihood of a successful venous puncture at the first attempt, thus reducing vascular damage. Also in our study, repeated unsuccessful attempts at inserting catheters increased the risk of catheter failure after successful placement. Multiple or difficult catheterization attempts often result in skin or venous bruising at the insertion site and may increase the incidence of phlebitis.^23^ Furthermore, repeated catheterization can cause pain, confirming the need to improve the first-time PVC insertion success rate.

This study and others demonstrated that irritant fluid infusion and drugs such as antibiotic use, vasoactive drug use, antihemorrhagic drug use, and dexamethasone use, have an impact on PVC failure.^16,20^ High concentrations of irritant fluids and drugs can increase plasma osmotic pressure, cause a fluid shift from within the vascular endothelial cells to the extracellular space, infiltration/extravasation, and vascular stiffness.

Our data suggested that prolonged duration of infusion and total daily infusion volume were risk factors for PVC failure. A previous literature review revealed that duration of infusion was a risk factor for extravasation and infiltration injuries.^22^ Infusing fluid for a long period of time exposes the vascular intima to forces that can cause tissue damage and an increase in vascular permeability, which can lead to infiltration/extravasation. We showed that the risk of PVC failure was increased at a total daily infusion volume of 501–1000 ml compared to a total daily infusion volume of <500 ml, a finding that is consistent with another study that reported a total daily infusion >1500 ml was a risk factor for phlebitis.^24^ However, we found the incidence of PVC failure did not increase when total daily infusion volume was ⩾1000 ml. This may be because the body produces an adaptive response to long-term stimulation of the vascular intima by larger infusion volumes rather than the acute inflammatory response that is evoked by smaller volumes and leads to PVC failure.

Also in this study, the use of heparinized saline for tube sealing compared to normal saline was associated with an increased risk of PVC failure. In accordance with these findings, a previous study showed that increasing concentrations of heparin were associated with increasing risk of phlebitis.^25^ The US INS guideline^14^ recommend the use of preservative-free 0.9% sodium chloride solution as a tube sealing solution in adult patients. A previous prospective, controlled study in patients with gastrointestinal or liver disease compared the effects of normal saline and heparinized saline for tube sealing. Findings showed no significant differences in the incidence of occlusion or other adverse events associated with PVCs in patients who received either normal saline or heparin saline.^26^

Based the results of the present study, we recommend that healthcare providers receive practical training on the venipuncture technique, avoid repeat puncture at the same insertion site, and avoid puncture sites that are associated with the risk of PVC failure. In particular, the site of PVC placement in elderly surgical patients should be continually monitored for early detection of PVC failure. Healthcare providers should have a list of drugs classified as irritants or vesicants so they can monitor patients receiving fluid infusions containing these drugs more frequently. Continuous PVC failures may result in the wrong dose of drug being delivered at the wrong time and vein wasting. An alternative device such as a mini midline, a midline, or a peripherally inserted central catheter (PICC) should be considered for patients receiving large infusions over an extended period of time or irritants or vesicants. Healthcare providers may consider using a vascular access management tool, such as the UK Vessel Health and Preservation (VHP) framework^27^ or the I-DECIDED clinical decision-making tool,^28^ for device selection and replacement or removal of a device.

Our data also showed that department (Obstetrics and Gynecology), catheter type (closed safety and open PVC), reinforced dressing, and indwelling period 48 h < *t* ⩽72 h, 72 h < *t* ⩽96 h, *t* > 96 h were independent predictors of PVC success. In this study, patients in the department of Obstetrics and Gynecology had a lower incidence of catheter failure compared to patients in other departments, possibly because patients in the department of Obstetrics and Gynecology require less intravenous fluids and are less likely to receive drugs due to concerns about fetal safety.^20^ With regard to catheter type, in contrast to our findings, a randomized control trial conducted in adult patients requiring a PVC on three medical and surgical wards at the Hospital Clínico San Carlos showed a 29% reduction in the incidence of phlebitis with closed safety versus open PVCs,^29^ while a second randomized trial confirmed that closed PVC systems are safer and more economical than open systems.^30^ Further research is required to understand the reasons behind these disparate results. With regard to reinforced dressing, the previous study in adult patients requiring a PVC in the medical and surgical wards of a tertiary hospital located in Queensland, Australia found no significant difference in the effect of reinforced dressings and ordinary dressings on the incidence of phlebitis.^16^ It is likely that a catheter that is not fixed properly may move in the blood vessel, damage vascular tissue, and cause complications.^31^ The discrepancy between the previous study and our findings may be resolved by additional investigations. With regard to indwelling period 48 h < *t* ⩽ 72 h, 72 h < *t* ⩽96 h, and *t* > 96 h, the previous study in adult patients scheduled for infusion therapy at one of two tertiary hospitals in Hunan China showed median dwell time to catheter failure was 52 h (interquartile range: 36–73 h), the incidence rate of catheter failure significantly increased by 1.1%/h in the first 38 h after catheter insertion, the incidence rate was significantly decreased from 39 to 149 h, and at >149 h, there was no significant change in the incidence rate.^20^ In the present study the incidence of PVC failure decreased at >48 h, possibly because the body mounted an adaptive response to the catheter as indwell time increased.

## Limitations

This study was limited as it did not consider the healthcare providers’ experience with the venipuncture technique and special consideration was not given to patients receiving infusions containing drugs classified as irritants or vesicants, which should be explored in future studies.

## Conclusion

This study should raise awareness among healthcare providers of the risk factors for PVC failure, including older age, hospitalization on the surgery or emergency/internal/surgical ward, history of venous puncture in the last week, forearm insertion site, ⩾2 puncture attempts, irritant fluid infusion, daily infusion time >4 h, daily infusion volume 501–1000 ml, and use of heparinized saline as the sealing liquid. Healthcare providers should use vascular access device selection tools that consider type of device, the nature and amount of the infusion, and the duration of the infusion to select a clinically appropriate device. During catheter indwelling, evaluation tools should be applied for the timely detection of complications. Healthcare providers should have a list of drugs classified as irritants or vesicants so they can monitor patients receiving fluid infusions containing these drugs more frequently.
